# Modern Medico-Psychology and Psychiatry

**Published:** 1895-02-02

**Authors:** T. S. Clouston

**Affiliations:** Lecturer on Mental Diseases, Edinburgh University, and Physician Superintendent Royal Asylum, Morningside


### MODERN MEDICO-PSYCHOLOGY AND PSYCHIATRY.

By T. S. CLOUSTON, M.D., Lecturer on Mental Diseases, Edinburgh University, and Physician Superintendent Royal Asylum, Morningside.

### XV.—STATES OF DEFECTIVE INHIBITION AND OF UNCONTROLLABLE IMPULSE.

(*Continued from p.* 253).

THE morbid impulses may be divided into 1, general impulsiveness in all directions; 2, epileptiform impulse; 3, animal and organic impulse; 4, homicidal impulse; 5, suicidal impulse; 6, destructive impulse; 7, drink impulse, or dipsomania; 8, kleptomania; 9, pyromania; and 10, immoral impulses, or "moral insanity." But these are only the chief *forms* of one disease; they do not constitute each *a disease* or distinct pathological entity by itself. There are very many lesser forms and varieties, such as *Planomania*, or an impulse to wander away without aim, and throw off the restraints of society; *Lycanthropia*, to act like a wild beast, and many more. Some authors have a tendency to multiply these as distinct forms of mental disorder. This I think entirely unscientific. They are really different symptoms of the one disease.

Many of these forms of morbid impulse or loss of control usually occur as symptoms of general insanity, while some of them may be met with apart from other mental disturbances. I must say I have never met with either pyromania or kleptomania by themselves as the only mental disturbance or weakness present in any case.

The causation of these morbid conditions of uncontrollable impulses is very varied. Morbid heredity is accountable for many cases; excessive drinking for many others; brain disease and brain injuries for others; brain exhaustion from child-bearing and nursing and loss of blood for others; the crises of life, such as puberty, the climacteric, and senility for others; and congenital weaknesses for others. It may be said that, given a certain quality of brain, whatever weakens it will set up morbid impulses and lessened control. Some people are angels or demons according as their brains are rested or tired. Which of us has the same control over his speech or temper when weak as when strong? The whole subject is a fascinating, but most difficult one, that yet needs much study and elucidation. Our knowledge of the physiology and psychology of the brain must advance much before we will be able fully to explain these departures from moral health.

While I cannot here go into the special symptoms and relations of all those forms of impulsiveness and losses of control, the subject of Dipsomania needs a more than passing notice. The drink craving is so lamentably common, is attended by such social ruin, and is imagined to be so markedly increasing in modern times, that the medico-psychological view of it must be shortly stated here. The drink craving cannot be separated from the cravings for morphia, cocaine, and chloral, and such brain stimulants and sedatives. The same quality of brain that leads to the one also leads to the others in different circumstances. But the alcohol craving is so much the most common that it must stand as the type of this kind of disease. Alcohol has the closest affinity for the nervous structures, especially the highest of them all found in the brain cortex. Its first action is on them, when it reaches them through the blood. In small quantities it unquestionably stimulates the mental powers, and produces pleasurable subjective sensations in most cases. In certain brains these effects are intensely agreeable, and the desire for a repetition of the dose is overmasteringly keen. Those are commonly just the persons who, through heredity or otherwise, have a small development of inhibitory power. Continued yielding to this desire for the alcoholic stimulus by and bye sets up a craving, while the most marked of the permanent effects of drink is to diminish the controlling centres of the brain. Thus, with an intensified craving and a diminished self-control, we soon arrive at a condition of brain in some cases where the inhibition against drink is practically lost —where, in fact, we have disease that must be treated by the doctor, and not merely self-indulgence or vice that can be safely left to the moralist or the magistrate. But the doctor cannot effectually treat or cure this complaint without interfering with personal liberty; we must have power forcibly to prevent the patient getting alcohol. Hence we need to go to Parliament. At present the law assumes that any British subject has the legal right to drink himself to death if he likes, while other forms of suicide are either criminal or can be treated and restrained under the provisions of the lunacy laws. This is one of the urgent questions at present for our profession, and much more so for society. The "liberty of the subject" idea is held up by lawyers and politicians as the bogey to prevent any effectual law with this object being passed. Doctors have nothing to do with ordinary drunkards, who can drink or give up drink when they choose, and they do not want to have anything to do with such persons. But they do desire, for the sake of society, to have power to treat a case of which the following is an aggravated type. I was consulted about a youth of fifteen, the son of an insane mother, and of a nervous constitution, who, after having had a glass of whisky given him by a companion, immediately developed an absolutely uncontrollable and diseased craving for the stimulant, which seemed to have awakened a sleeping demon within his brain. All his moral nature was overset. He lied; he stole; he was violent in personal conduct, and his affection for his relations was completely destroyed. This universal and complete breakdown of morality and affection is very characteristic of dipsomania. If ever a brain was in a condition of disease, it was that of this unfortunate boy. Yet, in the present state of the law, he could not be effectually treated towards recovery, because that would have implied interference with his liberty. Untold misery must be endured by families, social consequences of the most evil sort are happening in every grade of society, and individuals are deprived of their natural right to be cured of disease, because politicians have misapplied prejudices about personal liberty! The whole medical profession, who really have to do with the disease, and know its nature, unanimously demand legislation to help them to cure their patients; but, as yet, their representations to Parliament have been unavailing in this matter.

